# Hip Joint Replacement Using Monofilament Polypropylene Surgical Mesh: An Animal Model

**DOI:** 10.1155/2014/187320

**Published:** 2014-05-29

**Authors:** Jacek Białecki, Marian Majchrzycki, Antoni Szymczak, Małgorzata Dorota Klimowicz-Bodys, Edward Wierzchoś, Krzysztof Kołomecki

**Affiliations:** ^1^Department of General Surgery, Franciszek Raszeja Memorial Hospital Poznan, Mickiewicza 2, 60-834 Poznan, Poland; ^2^Department of Rheumatology and Rehabilitation, Poznan University of Medical Sciences, 28 Czerwca 1956 Roku 135/147, 61-545 Poznan, Poland; ^3^Department of General Surgery, Medical Center H.C.P. Poznan, 28 Czerwca 1956 Roku 194, 61-485 Poznan, Poland; ^4^Veterinary Clinic, Kozanowska 40/16, 54-152 Wroclaw, Poland; ^5^Department of Swine and Small Ruminant Breeding, Faculty of Animal Breeding and Biology, University of Agriculture, Mickiewicza 24/28, 30-059 Krakow, Poland; ^6^Department of Endocrine, General and Vascular Surgery, Copernicus Memorial Hospital in Lodz, Medical University of Lodz, Pabianicka 62, 93-513 Lodz, Poland

## Abstract

Hip joint dysplasia is a deformation of the articular elements (pelvic acetabulum, head of the femur, and/or ligament of the head of the femur) leading to laxity of the hip components and dislocation of the femoral head from the pelvic acetabulum. Diagnosis is based on symptoms observed during clinical and radiological examinations. There are two treatment options: conservative and surgical. The classic surgical procedures are juvenile pubic symphysiodesis (JPS), triple pelvic osteotomy (TPO), total hip replacement (THR), and femoral head and neck resection (FHNE). The aim of this experiment was to present an original technique of filling the acetabulum with a polypropylene implant, resting the femoral neck directly on the mesh. The experiment was performed on eight sheep. The clinical value of the new surgical technique was evaluated using clinical, radiological, and histological methods. This technique helps decrease the loss of limb length by supporting the femoral neck on the mesh equivalent to the femoral head. It also reduces joint pain and leads to the formation of stable and mobile pseudarthrosis. The mesh manifested osteoprotective properties and enabled the formation of a stiff-elastic connection within the hip joint. The method is very cost-effective and the technique itself is simple to perform.

## 1. Introduction

Hip joint dysplasia is classified as a developmental trait and occurs mainly in medium- and large-breed dogs, rarely in small-breed dogs and cats. It is a deformation of the articular elements (pelvic acetabulum, head of the femur, and/or ligament of the head of the femur) leading to laxity of the hip components and dislocation of the femoral head from the pelvic acetabulum (subluxation or luxation) and, in consequence, to hip joint degeneration resulting in joint pain and lameness [1–3].

The etiology of hip dysplasia is not fully understood. It is assumed that genetic factors play a decisive role and disease intensity and early development of clinical symptoms are amplified by environmental factors [4, 5].

Diagnosis is based on symptoms observed during the clinical examination (Ortolani test, the Barlow manoeuvre, Barden's manoeuvre, evaluation of the animal's posture, pelvic shape, and gait) and imaging studies [6–8].

In general, two treatment options exist: conservative and surgical. The former is applied in older, severely diseased animals or when consent for surgical treatment cannot be obtained. It involves pain reduction and preservation of limb function allowing for normal physical activity. The surgical procedures currently available are continually being improved and include the following techniques: triple pelvic osteotomy (TPO) [1, 9, 10], juvenile pubic symphysiodesis (JPS) [6, 7, 9], femoral head and neck resection (FHNE) [11], or total hip replacement (THR) [12–14]. Many of these surgical procedures are highly successful with relatively low complication rates; however, these techniques are expensive and require a long and gradually increasing rehabilitation program in order to restore full fitness. Recovery is frequently hampered by pain in the operated area.

The presented paper suggests a new surgical technique utilising monofilament polypropylene hernia mesh for partial replacement of the femoral head.

## 2. Material and Methods

This study was carried out by the Department of Swine and Small Ruminant Breeding at the University of Agriculture in Krakow and Department of Animal Physiology and Biostructure at the University of Environmental and Life Sciences in Wroclaw. This experiment was conducted in accordance with Polish legal requirements for animal welfare and experimentation. The study was approved by the Local Bioethics Committee Poznan, Poznan University of Life Sciences (approval number 79/2011).

### 2.1. Animals and Operating Technique

In this study, six lambs (Polish Lowland Sheep) weighing 12–15 kg (18 weeks old) and two adult Heath sheep weighing 35 kg (3 years old) were used.

Twenty-four hours prior to surgery the sheep were starved by restricting nutritive and bulk fodder, but the access to water was available. Twelve hours prior to surgery, the access to water was also restricted. Both groups (adult sheep and lambs) were premedicated with atropine sulfate (0.2 mg/kg i.m.) and xylazine hydrochloride (0.25 mg/kg i.v.). Subsequently, they were shaved and the surgical site was prepared. The area was additionally anaesthetized with 2% lidocaine. General anaesthesia was induced using ketamine hydrochloride (30 mg/kg i.v.). Metamizole sodium (30 mg/kg i.v.) and infusion fluids were administered.

A curved incision, 10 cm long, was made over the femoral head to gain surgical access. The hip joint capsule with ligaments was then incised and the femoral neck was exposed. The femoral head ligament was then cut and the femoral head was separated from the femoral neck using Liston forceps and removed from the articulation area.

**Figure 1:**
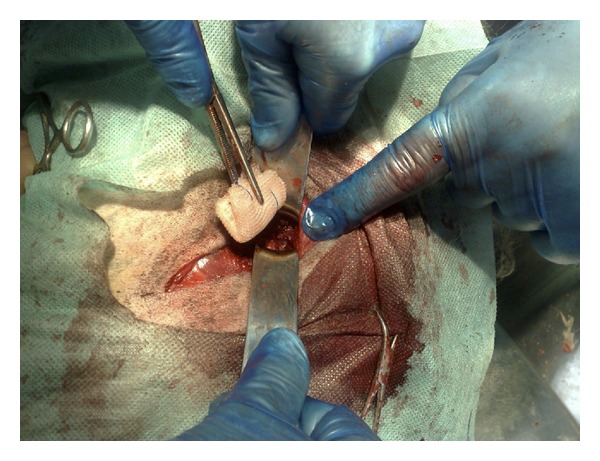
Intraoperative photograph demonstrating introduction of polypropylene mesh into the acetabulum.

A monofilament propylene hernia mesh folded repeatedly, commonly used in the alloplasty of inguinal hernia in humans (surgical mesh 15 × 15 cm, Grena Ltd., East Sussex, UK), was introduced into the hip joint, replacing the removed femoral head (Figure 1). After measuring the diameter of acetabulum, flat mesh folded into a cube was placed so it filled the acetabulum entirely (around 4 × 3 × 3 cm).

**Figure 2:**
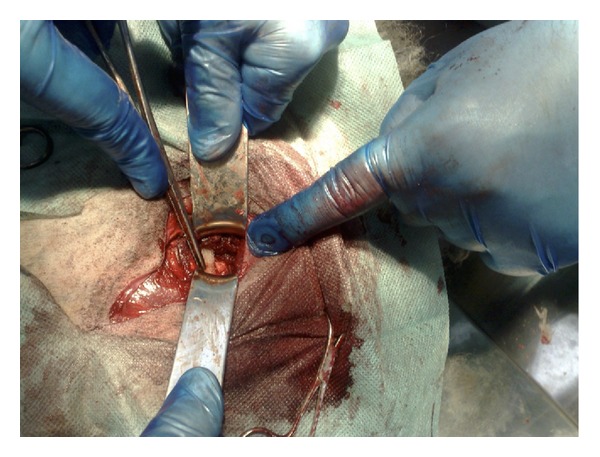
Final view of the “actual” acetabular component in situ.

The size of the folded mesh was adapted to each acetabulum. The surgical mesh was placed in the acetabulum stabilizing the femoral neck resting on it (Figure 2).

After surgery the sheep were isolated in separate boxes, where they received a weight-adjusted amount of nutritive fodder, hay, and unlimited access to water. The operative wound was assessed on the day following surgery. A combination of penicillin G and dihydrostreptomycin at 1 mL/10 kg dosage and metamizole sodium were given intramuscularly every 24 hours. A vitamin supplement (Catosal 10%, Bayer Animal Health) was also administered daily. Treatment continued till Day 4. The stitches were removed on Day 7 or Day 8. On Day 14 the sheep were allowed to join the herd in the pasture, where the subsequent clinical evaluation took place. In the twelfth postoperative week the animals were euthanized.

### 2.2. Radiographic Examination

Control radiograms of the operated joint were obtained in the eleventh postoperative week. For the evaluation of the hip joint, Ortolani's manoeuvre and extended-hip radiography were used. To obtain these views, the sheep were placed on their backs in dorsal recumbency with the rear limbs extended and parallel to each other [15].

### 2.3. Histological Examination

The bone samples for the histological examination were fixed in 40% buffered formalin for 14 days. Then the samples were dehydrated in the following baths: 10% solution of disodium EDTA for 21 days and a mixture of formic acid and sodium citrate for 28 days. Subsequently, the samples were embedded in paraffin wax. Sections, 6.0 to 7.0 *μ*m thick, were made and stained with Delafield's haematoxylin and eosin for light microscope evaluation.

## 3. Results

### 3.1. Clinical Examination

The wound was evaluated in the first postoperative day and no indication of inflammation was observed. The healing of the surgical wounds was normal in each animal. The operated limb was not loaded. On the fifth postoperative day, the animals did not yet stand on the operated limb (grade 3 lameness). However, no increased pain was observed. The first attempts to load the operated limb were observed.

After 14 days the animals joined the herd in the pasture. Full loading of the limb and its use for support, accompanied by slight lameness, were then observed (grade 2 lameness). Full loading of the extended limb was achieved in approximately the fourth week. After eight weeks complete stability in the operated joint with full limb loading was observed. However, lameness was still noted (grade 1 lameness). It must be emphasised that the sheep in the pasture were required to move in order to reach the water trough and shelter in the sheepfold overnight. No extended periods of lying were observed during the time spent in the pasture. All animals behaved the same during the evaluation of lameness.

**Figure 3:**
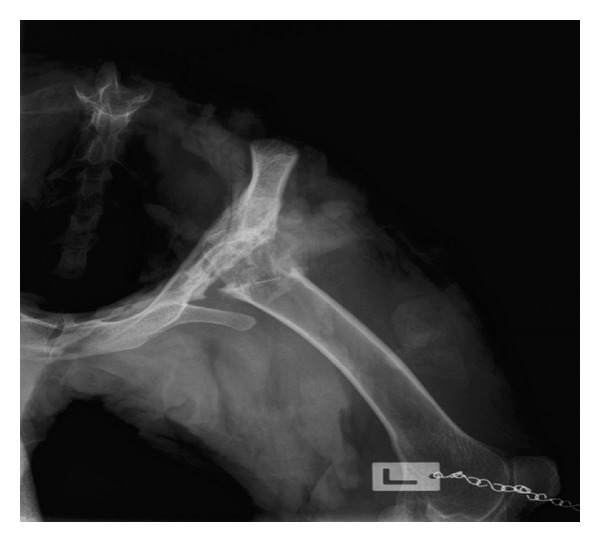
Sheep pelvis following alloplasty of the left hip joint.

The localisation of the mesh implant within the acetabulum is presented in Figure 3. The forming osseous tissue develops on both the sides of the neck and acetabulum, creating a stiff connection with the implanted mesh. The remaining part of the implant, with no gathering of osseous cells, maintains its elastic properties. It allows for partial mobility in the hip joint, with simultaneous compactness. This was confirmed during the clinical evaluation of the operated animals.

After the surgical mesh was placed in the acetabulum, stabilizing the femoral neck resting on it, there was no direct damage to the acetabulum by the neck, and the distance between the acetabulum and the distal femoral epiphysis remained unchanged. The limb's length was preserved. The procedure ended with one-layer stitches of the articular capsule and muscles and single vertical mattress stitches of the skin.

### 3.2. Radiological Evaluation

**Figure 4:**
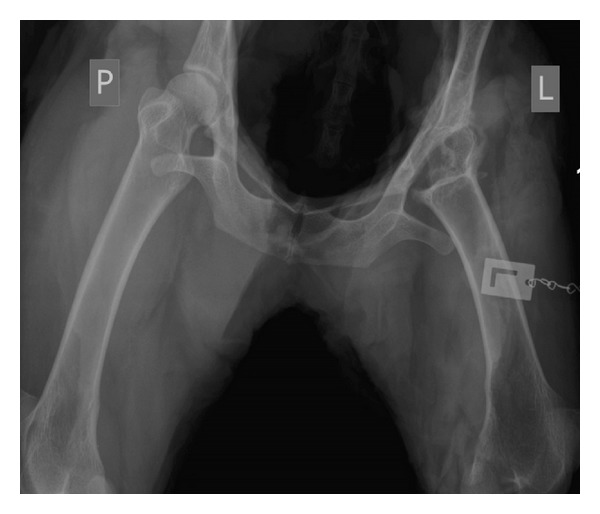
Normal (right, P) and postalloplasty (left, L) hip joint in the sheep.

An X-ray of the operated hip joint was obtained in the eleventh postoperative week and no radiological signs of joint inflammation or dislocation of osseous structures were observed. Radiography of normal (right, P) and postalloplasty (left, L) hip joint in the sheep is presented in Figure 4. In this period, the animals moved freely over the pasture area, fully loading the operated limb, with partial movement in the hip joint. The asymmetrical image on the X-ray shows how the mesh implant in the left hip joint behaves as opposed to the right hip joint which was not operated on.

### 3.3. Histological Evaluation

**Figure 5:**
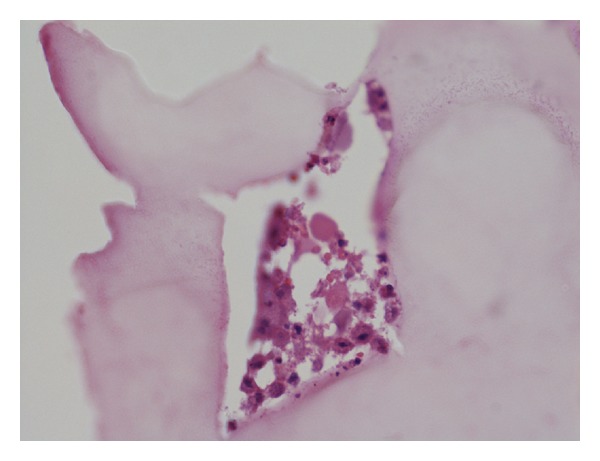
Histological picture of ongoing osteogenesis after mesh implantation.

The performed histological examinations demonstrate the formation of osseous tissue on the surface of the fibres of the polypropylene mesh, as shown in Figure 5. The cells adhering to the surface of the polypropylene mesh fibres are mainly osteoblasts, whereas some fibroblasts have also been noticed. A matrix is observed between the cells. The histological picture indicates ongoing osteogenesis.

## 4. Discussion

Many surgical techniques improve the biomechanics of the diseased joint, reducing or totally eliminating clinical symptoms for a given period of time.

Knitted surgical polypropylene mesh is specifically designed for tissue reinforcement in hernia repair, eventrations, and prolapses through laparoscopic or conventional laparotomy surgery in humans. Polypropylene is chemically neutral and very well tolerated by tissue without inflammatory reaction. We decided to use monofilament polypropylene surgical mesh not only to minimize complications and injuries after surgery but also to minimize the cost of the treatment. Many surgical procedures are highly successful with relatively low complication rates; however, these techniques are expensive and require a long and gradually increasing rehabilitation program in order to restore full fitness. Recovery is frequently hampered by pain in the operated area.

The most common surgical techniques are triple pelvic osteotomy (TPO), juvenile pubic symphysiodesis (JPS), femoral head and neck resection (FHNE), and total hip replacement (THR).

Triple pelvic osteotomy is a procedure which involves cutting the pelvis in three sites (triple osteotomy), rotation of the acetabulum, and lowering of its vault for better covering of the femoral head. The osteotomy sites are then secured with special plates. TPO is a preventive management and candidates for this treatment should be less than 1 year of age, with no inflammatory or degenerative changes. This technique is recommended when hip dysplasia is diagnosed at an early stage, before secondary degenerative changes have occurred [1, 9, 10]. TPO is probably ineffective when there is a degenerative joint disease. Reported complications after TPO are related to pelvic anatomy, excessive stress placed on the implants, or both. Frequent deleterious effects are femoral dysplasia, obstipation, dysuria, and neurological abnormalities. Poor surgical technique may lead to complications such as iatrogenic injury to the sciatic nerve, progression of degenerative joint disease, and infection [16].

Juvenile pubic symphysiodesis is a minimally invasive surgical procedure without internal fixation devices and involves mechanic destruction of the pubic symphysis and its fixation in position, allowing the pelvic acetabulum to cover the femoral head [6, 7, 9]. It seems that this treatment produces better results in animals with less advanced changes in the hip joint. Reported complications after JPS may be subluxation or the development of osteoarthritis. In severe cases of canine hip dysplasia acetabular ventroversion occurs slowly and the femoral head slips laterally along the sloped and rounded lateral acetabular border, so acetabular congruity is not obtained [17].

Femoral head and neck excision is a procedure intended to significantly reduce pain associated with movement of an injured or diseased coxofemoral joint. Functioning of animals after FHNE is better in small dogs or cats compared with large dogs, because the ability to compensate for the mechanical disadvantages of an absent coxofemoral articulation is dependent on weight. It has been suggested that the maximum weight limit is 17 kg, above which muscle competency for maintenance of a functional and durable pseudarthrosis is unlikely to be established. This surgery has been performed in dogs for over 30 years with success rates as high as 98%. Common complications after this procedure, noted by owners and on clinical examination, include pain caused by the femoral neck mechanically irritating the acetabulum [11], lameness associated with limb shortening, patellar luxation, sciatic neurapraxia, and also severe muscle atrophy which results in limited hip motion range [12].

Total hip replacement is commonly used and widely accepted as a surgical procedure for painful, irreversible, developmental, or acquired conditions of the coxofemoral joint, especially in large dogs. It is a treatment for severe or refractory conditions of canine hip joint, with a wide range of both cemented and cementless implant systems. This method is associated with high success rates (92–98%) and relatively low complication rates (7.8–20%), based on both owner assessment and clinical evaluation of pain status and functionality [14]. Reported complications after THR in dogs include luxation/dislocation, septic loosening, aseptic loosening of both cemented and cementless components, improper implant positioning, periprosthetic femoral fractures, sciatic neurapraxia, patella luxation, extraosseous cement granuloma formation, and femoral medullary infarction [13]. The clinical value of hip replacement procedures is questionable, due to the high cost of the implant which must be individually manufactured and the high complication rates associated with this technique [14].

No data concerning the use of polypropylene mesh as an equivalent of the femoral head in the treatment of hip joint dysplasia and degeneration was found in the available literature. The goal of this experiment was to present an original technique of filling the acetabulum with a polypropylene implant, with the femoral neck resting directly on the mesh. The goal is to reduce the loss of limb's length by supporting the femoral neck on the mesh equivalent to the femoral head and also to reduce joint pain and to lead to the formation of stable and mobile pseudarthrosis. It should be noted that this surgical technique is straightforward. The introduction of the multiply folded mesh and adjusting it to the size of the acetabulum are simple and do not require any special skills from the surgeon.

The clinical value of the method was positively verified. The clinical assessment in the second postoperative week did not show any increased need for analgesics. First attempts to load the operated limb were then made and gradually increased till full loading was achieved in the fourth postoperative week. Despite observed lameness, the stability of the lower limb was full, with preserved partial mobility. It is worth emphasising that despite the implantation of foreign material, that is, polypropylene mesh, wound healing was uneventful (primary intention).

The polypropylene mesh, acting as an equivalent for the femoral head, enabled formation of a stiff-elastic connection within the hip joint. The radiograms performed in the eleventh postoperative week revealed the formation of osseous tissue at the femoral neck and side of the mesh, whereas no such ossification was observed on the inner side of the implant. It may therefore be assumed that the image confirms the stability of the neck-mesh connection with concomitant preservation of partial elasticity of the mesh implant (according to the stiff-elastic model presented in the above-mentioned scheme). The histological examination of the operated bones revealed the osteoprotective properties of the polypropylene mesh used in the study. The histological evaluation confirmed ongoing osteogenesis within the polypropylene mesh, especially at the femoral neck end.

## 5. Study Limitation

This was a pilot study and the authors wanted to introduce a new surgical technique in animals. There was no control group which would undergo the same surgical procedures but without the polypropylene mesh.

The authors did not make the measurements of the operated limbs, because without the control group the absolute values would not be reliable.

There are plans to expand this project further in the future, using larger number of animals and a control group.

## 6. Conclusion

This paper is a report on the use of polypropylene mesh in orthopedics. Until recently it has only been used in the surgical treatment of abdominal hernia, where it is used as fascia implant. In hernia surgery, polypropylene mesh is well tolerated by patients, leading to permanent recovery with low rate of postsurgery complications. Hernia repair is considered by the European Hernia Society to be a treatment of choice (a recommended procedure). Guided by these references we decided to use this implant in bone surgery. On the basis of clinical evaluation of operated animals, we tentatively can say that the mesh was well tolerated by them. Histological examination showed osteoprotective properties of polypropylene mesh.

We expect that further studies will confirm the osteoprotective effect of polypropylene mesh in orthopedics.
